# EEG as an indispensable tool during and after the COVID-19 pandemic: A review of tribulations and successes

**DOI:** 10.3389/fneur.2022.1087969

**Published:** 2022-12-02

**Authors:** Brin E. Freund, Anteneh M. Feyissa

**Affiliations:** Department of Neurology, Mayo Clinic Florida, Jacksonville, FL, United States

**Keywords:** COVID-19, electroencephalography, epilepsy, pandemic, rapid EEG, seizure, Tele-EEG

## Abstract

During the coronavirus disease 2019 (COVID-19) pandemic, elective and non-emergent tests and procedures were delayed or suspended in lieu of diverting resources to more emergent treatment of critically ill patients and to avoid the spread and contraction of COVID-19. Further, the workforce was stretched thin, and healthcare facilities saw high turnover rates for full-time and contract employees, which strained the system and reduced the ability to provide clinical services. One of the casualties of these changes was electroencephalography (EEG) procedures, which have been performed less frequently throughout the world since the pandemic. Whether considered routine or emergent, the deferral of EEG studies can cause downstream effects, including a delay in diagnosis and initiation of treatment for epilepsy and non-epileptic seizures resulting in a higher risk of morbidity and mortality. Despite these limitations, the importance and utility of EEG and EEG technologists have been reinforced with the development of COVID-related neurological complications, including encephalopathy and seizures, which require EEG for diagnosis and treatment. Since the pandemic, reliance on remote telemonitoring has further highlighted the value and ease of using EEG. There has also been a heightened interest in rapid EEG devices that non-technologist professionals can attach quickly, allowing minimum patient contact to avoid exposure to COVID-19 and taking advantage of remote EEG monitoring. This review discusses the acute and potential long-term effects of the COVID-19 pandemic on the use and performance of EEG.

## Introduction

The coronavirus disease 2019 (COVID-19) pandemic posed unprecedented challenges and stressed the healthcare system, with the fallout still being felt nearly 3 years after it began. Significant changes occurred as healthcare providers and facilities adjusted to the growing need for emergent and intensive care treatment, with medical floors and intensive care units filled with COVID-19 patients. The direct impact of the pandemic on healthcare has been well-described. It includes reduced and reconfigured clinical services, particularly those deemed non-urgent and, or elective, and an increase in the use of telemedicine (1–3). Many of these adjustments were driven by concerns for spreading infection, financial constraints, closure of health care facilities and offices, and reduced staffing (1, 4), disproportionately affecting the most vulnerable in society (5), including patients with epilepsy (PWE).

Given the efforts focused on providing care to those with COVID-19 infection, there was a de-emphasis on outpatient and inpatient evaluations of seizures involving newly referred patients (6–14) and those with chronic epilepsy (3–5, 10, 15, 16) leading to difficulty in providing care to both adults and children (3, 5, 11). One to two-thirds of epilepsy care providers reported that the pandemic had negatively impacted their practice (4, 10). Besides, roughly 25–50% of PWE and their caregivers reported difficulty accessing care (3, 10, 17), and there was a 25% reduction in outpatient visits at epilepsy centers (12).

These limitations and restrictions not only pertained to in-person consultations but also included electroencephalogram (EEG) studies with a significant impact on clinical care, given that roughly 4,000 routine EEGs are performed annually at many epilepsy centers (18). Despite the importance of EEG in assessing newly diagnosed acute seizures and encephalopathy, which are commonly seen in COVID-19-infected patients, and in monitoring patients for pre-surgical and diagnostic evaluations, reports worldwide indicate reduced access to inpatient and outpatient EEG services, including long-term video EEG monitoring (LTVEM) in epilepsy monitoring units (EMUs) during the COVID-19 pandemic (6–12). The restricted access to EEG has had direct and measurable effects, with the downstream medium and long-term consequences still to be determined.

This review discusses the difficulties and challenges of providing care for PWE and those with newly diagnosed seizures during the COVID-19 pandemic, focusing on EEG service lines. We also describe some of the modifications and advancements in EEG that arose from the pandemic's adverse conditions. Besides, we consider the future as a result of these tribulations and successes as they will affect the processes and uses of EEG.

## Trials and tribulations

Despite being a highly utilized procedure, EEG studies had to be suspended or limited during the COVID-19 pandemic, which expectedly had many direct and indirect effects on the care of patients with seizures. However, interest in EEG increased during the pandemic, given the neurological conditions associated with COVID-19 that required EEG for evaluation (19). This led to competing motivations, whereby EEGs were ordered for clinical purposes but could not be performed. Challenges arose as a result of these disparate concerns.

Understanding the mechanisms by which EEG was restricted is crucial in adapting clinical practice and limiting these changes' effects on patient care. Governmental and institutional regulations led to the diversion of clinical resources to support COVID-19 initiatives. There was also a reluctance to perform EEGs and transfer patients between hospitals to avoid contracting and spreading COVID-19 to EEG staff (6, 20). The reduced inter-hospital transfers impacted the use of EEG. Many facilities often refer patients to larger centers for inpatient EEG and continuous EEG (cEEG) monitoring that are unavailable at their institution (6). Further, expert recommendations specifically addressed the need to avoid urgent evaluations for non-urgent testing, which included diagnostic EEG (21). These changes had a disproportionate effect on low and middle-income countries (10). Table 1 summarizes the trials and tribulations during the pandemic.

**Table 1:** Success and trials and tribulations of EEG service during the COVID-19 pandemic.

**Trials and tribulations**
Restrictions on performing routine outpatient EEG
Inpatient EEG performed only on urgent bases and routine studies discouraged
Reduced inter-hospital transfers affecting inpatient and continuous EEG
Reduced EEG performance associated with worse outcomes
Routine outpatient EEGs deferred in patients with symptoms or recent COVID-19 infection
Increased use of EEG in emergency departments leads to strain on emergency services
Reduction and often suspension of EMU admissions
Cancellation of adult and pediatric EMU evaluations
EEG technologist shortage further strained by the reorganization of healthcare systems
**Successes**
Best practice protocols, including equipment disinfection and allocation
Standardization of triage for inter-hospital transfers and teleconsultations
Protocols set in place for a more streamlined EEG workflow
Increased use of rapid EEG systems
Increased use and advancements in Tele-EEG
Increasing use of ambulatory EEG
Incorporating smartphone videos into epilepsy care
Reassessment of standard EEG procedures
Emphasis on the importance of training and retaining EEG staff

### Routine inpatient and outpatient EEGs

Though EEG restrictions were in place to promote the safety and wellbeing of workers and patients, epilepsy care providers viewed limited access to EEG as being associated with worse patient outcomes (22). Roughly 50% of epilepsy care providers reported that inpatient EEGs in those with COVID-19 were discouraged, and 22% performed fewer EEGs overall (4). Many centers worldwide stopped performing EEGs at the height of the pandemic, both for clinical and research purposes, or limited its use to urgent evaluations (9). Decreased or delayed use of EEG occurred in many cases of new-onset seizures in outpatient (11) and inpatient settings (23). Outpatient EEGs were reduced by at least 32% in Japan in 2020 (14). Others reported that if EEG was performed, only patients who did not have symptoms or recent infection with COVID-19 were allowed to be evaluated (9). Reports on inpatient EEG studies, including cEEG, have been varied, with most noting a reduction by at least 50% (6–10) and fewer noting an increase in studies (4, 24). On the other hand, there has been an increased use of EEG in the emergency department, likely related to difficulties in providing clinical and EEG services in outpatient settings, further straining emergency care services (25). Many of these effects were felt by both adult and pediatric neurologists. One study reporting on the opinions of pediatric epilepsy care providers noted that 90.6% had reduced access, 3.6% had no access, and 13.5% with inpatient access only to EEG services (11). In a pediatric epilepsy center, 76% of outpatient EEGs ordered in outpatient settings were canceled in response to local restrictions on healthcare services during the Pandemic (16).

### Long-term video EEG monitoring

LTVEM in epilepsy monitoring units EMUs was also significantly reduced and even suspended at many facilities for some time during the pandemic (4, 11, 12, 26) with an aggregate decrease of 23% in all National Association of Epilepsy Centers (NAECs) (12). European inpatient EMU and LTVEM services were restricted in 38.3% of cases involving adults and 53.2% of children and stopped altogether in 61.7% of adults and 36.2% of children (8). If admissions were allowed conditionally, they were usually limited to urgent or life-threatening cases, as opposed to elective, standard pre-surgical, or diagnostic evaluations (11, 27). This led to the inability to optimize medical therapies in complex epilepsies and complete the pre-surgical work up for planning epilepsy surgeries at many institutions (8), which unnecessarily placed patients with drug-resistant epilepsy at an increased risk of mortality, including sudden unexpected death in epilepsy (SUDEP) (14, 28–30). This also likely affected recent onset functional seizure disorders as many could not be evaluated with LTVEM in an EMU setting due to COVID restrictions, likely leading to underdiagnosis and misdiagnosis (31). Restrictions on EMU admissions and subsequent delays in diagnosis and treatment could have other implications, including a higher risk of suicide in those with functional seizures as well as increased rates of morbidity and mortality in those with non-epileptic spells related to physiological disorders, such as cardiac dysrhythmias that may also be diagnosed in the EMU during admission (32).

### Epilepsy surgery

Although delays or deferrals in evaluations in epilepsy clinics and EMUs result in downstream effects on diagnosis and fewer referrals for epilepsy surgery and invasive EEG recordings (8), hospitals limited epilepsy surgery at the height of the pandemic in order to divert resources from elective procedures to emergent treatment of COVID-19 (10, 14). These effects of the pandemic further added to long waiting lists for epilepsy surgery and delayed the chance of seizure freedom (10). Nearly 70% of European centers reported that epilepsy surgery was not being performed, while most other institutions were working at a reduced capacity (8). Invasive EEG was also stopped in 80% of European epilepsy centers, with only 3.3% reporting no change in its performance (8). In an international report on pediatric centers, 91.3% had either reduced or halted epilepsy surgeries during the pandemic (11). The effects were more modest in North America. In a report on NAECs, VNS implantations were reduced by 19% overall at level 4 and adult centers, and extratemporal resections were significantly reduced in some facilities, while invasive EEG increased by 8.7%. Overall, there was a 5.7% reduction in surgical treatments, which may have been related to classifying some epilepsy surgeries as “non-elective” given the risks of deferring treatment (12).

### Shortage in EEG technologists

The dearth of well-trained EEG technologists was apparent during the pandemic, with the subsequent reduction in staffing of clinical EEG services to support the response to the COVID-19 pandemic (6, 20, 33). Prior studies before the pandemic noted a demand for nearly 40,000 jobs for neurodiagnostic technicians in the future and a lack of sufficient training programs to meet this need (34), and the pandemic exacerbated this problem. Given the toll taken on technologists as the stress of the pandemic worsened, these staff shortages were intensified and likely will continue into the future (35).

## Advancements and opportunities

Given the increased focus on providing care from a distance and the reliance on telemedicine, EEG remained a valuable clinical tool despite limitations in testing during the pandemic. Despite replacing in-person visits with remote encounters, there appeared to be little impact on ordering practices regarding EEG (16). Further, the value of remote EEG monitoring during the pandemic was demonstrated by its use in evaluating the neurological complications from COVID-19 (19). These successes are highlighted in Table 1.

### EEG workflow

Given the sustained requests for EEG services despite restrictions on its performance, including EMU referrals, efforts were made to improve processes and utilize technological advancements to better apply EEG technology to patient care while considering these limitations. New best practice protocols for performing EEG were proposed, including but not limited to disinfection and maintenance of equipment, proper personal protective equipment (PPE) use by patients and staff, the designation of machines to be used in ICU and COVID-19 units, and technologist staffing and safety (8, 9, 21, 26, 32, 33, 36– 44)^1^. Tertiary care facilities and especially those designated as epilepsy centers with EMUs were forced to create more streamlined and standardized triage protocols for intra-hospital and inter-hospital transfers and coordinating care with outlying hospitals, including teleconsultations (26), leading to safer practices and quicker reinstitution of services (45). Triaging recommendations were also formalized regarding when to order EEG to limit unnecessary testing and risk of exposure to staff and patients, explicitly pointing out that inpatient EEG should be performed mainly to evaluate for subclinical seizures and unexplained mental status changes (33). As more studies were published on EEG in COVID-19-associated encephalopathy and seizures, it became evident that standardized protocols were needed to appropriately order and use EEG and cEEG even in a non-restricted setting outside of the Pandemic (40, 46, 47).

### Rapid EEG

Given the increased risks of infection from EEG technologist setup in those with COVID-19 infection, remote monitoring techniques became more important to limit exposure to critically ill patients while still providing EEG services. The importance of continuing to perform EEG was further supported by the reports from some centers on increasing referrals for cEEG related to concerns about non-convulsive seizures in critically ill COVID-19 patients (20) and providing neurological assessments in those who are sedated and paralyzed due to acute respiratory distress syndrome (ARDS) (48). The management of sedatives and anesthetics is particularly challenging in those with ARDS due to COVID-19, given the prolonged course of sedation, making bedside clinical evaluations difficult and, at best intermittent, leading to a higher risk of oversedation and delirium and prolonged ICU stays (48, 49). Further, there may be a discrepancy between bedside scoring paradigms (i.e., Richmond Agitation and Sedation Scale) and neurophysiological measurements of clinical sedation, which could contribute to oversedation and prolonged intubation with worsened outcomes (50). To enhance monitoring of sedation and to evaluate for non-convulsive seizures performing a standard cEEG is ideal, but given the labor intensiveness and concerns about transmitting infection, as well as obtrusiveness of standard EEG leads in COVID-19 patients who are in a prone position and intubated, proposals were made to try to incorporate technological advancements (48). This included using reduced montage EEG or rapid EEG devices, such as wireless wearable miniature EEG sensors, as well as relying more on telemetry and computer-based automated analyses and processed EEG for continuous neuromonitoring (50, 51).

Before the COVID-19 pandemic, there was a surge in studies of novel EEG sensor and acquisition options, including reduced montage and rapid EEG devices, which have also demonstrated efficacy during the pandemic (51–56). The pandemic has led to the broader use of these newer methods of EEG recording. They often use disposable electrodes, are easier to clean and maintain, and can be applied quicker, lowering the risk of transmitting infection (9, 24, 40). One study reported a reduction in setup time by 24 min with the use of reduced montage EEG, which was further aided by allowing for remote EEG acquisition during setup to be performed partially outside of the patient room with a laptop (24). Another report demonstrated a reduction in the overall setup time from 99 to 51 min, attributed to the reduced time to apply the reduced number of electrodes and lack of collodion (33). Rapid and reduced montage EEG studies were also implemented to successfully overcome the lack of technologist availability (20).

### Tele-EEG

Remote monitoring was also utilized in standard EEG montage studies performed for LTVEM outside the hospital. EMU admissions were also impacted during the pandemic, which led to system changes and the development of protocols improving the safety of patients and healthcare workers and, subsequently, the reinstitution of LTVEM in the EMUs in some centers (45). However, other methods of evaluating patients in the outpatient setting were also utilized, given the restrictions on inpatient elective admissions, with an emphasis on ambulatory EEG (26, 57). There have been previous studies demonstrating the utility of ambulatory EEG, which today uses similar technology to the inpatient EMU and can also include video recordings (57–59). This can be a safe and effective alternative to admission for LTVEM in the EMU, as long as proper disinfection of equipment is performed (57). Smartphones have also been utilized during the pandemic, with validity demonstrated as a diagnostic tool in recording epileptic and non-epileptic seizures for expert review (60–62). Given their efficiency and convenience, novel ways of performing diagnostic evaluations will continue to be used in the future.

### Innovations in EEG recording techniques

The value of routine procedures performed during EEG, such as hyperventilation, was reassessed, given the risk of producing aerosols and spreading COVID infection. Previous studies have demonstrated that hyperventilation has a low yield in eliciting focal interictal epileptiform discharges (63), though it remains a part of the standard operating procedure in many EEG laboratories. This is important, especially considering that hyperventilating with a mask applied produces different physiological effects that can impact the results of EEG testing (64). As a result, when weighing the risks and benefits of performing hyperventilation with or without masking, studies concluded that hyperventilation could be withheld other than in cases of suspected generalized epilepsies, including childhood absence epilepsy (36) and juvenile myoclonic epilepsy (65), or exchanged for a complimentary maneuver (36). Though omitting hyperventilation in EEG can be considered in a pandemic, it may not be appropriate for precise diagnosis.

EEG electrode placement was also re-evaluated in the setting of the pandemic. Given the habituation of many nasopharyngeal swabs to test for COVID-19 infection during the pandemic, the study of nasopharyngeal electrodes was revived. They were found to be valuable in assessing interictal epileptiform discharges, which may lead to a resurgence in their use, given the relative ease of attachment compared to other accessory electrodes, such as those used for sphenoidal monitoring (66, 67).

### Investing in the future technologists

The importance of highly trained staff was also highlighted during the pandemic, as they could troubleshoot and perform clinical duties such as setting up EEGs more quickly and efficiently, thereby reducing the risk of exposure and transmission of the virus (33). The Importance of EEG technologists was further demonstrated in some centers that experienced increased requests for EEG during the pandemic, particularly cEEG in the inpatient setting (24).

## What does the future hold?

Though we are still in the woods regarding the COVID-19 pandemic, there has been a significant reduction in caseloads, and restrictions have eased considerably. We are now looking at the “medium” term effects the pandemic has had on the treatment of seizures and management of PWE, with some concerns regarding access to care still being raised, particularly regarding the delay in performing EEG and LTVEM in EMUs (68). It is also time to consider the long-term effects of the constraints placed during the pandemic, especially regarding EEG and LTVEM (27). Limitations on EEG and EMU evaluations in epileptic and non-epileptic seizure disorders will be expected to lead to higher morbidity (69) and, in drug-resistant epilepsy, a higher mortality rate and SUDEP (70). The impact will likely take years to determine as data is accumulated and these effects take hold. However, it will be imperative to continue to monitor these trends so we can better understand the effects of the pandemic on long-term outcomes in epilepsy and functional seizures to inform us of the importance of maintaining these vital clinical services in the future.

Despite the outlook regarding the effects of the pandemic, there were improvements and advancements that we should continue to explore and expand upon. The protocols set in place regarding infection prevention, as well as the use of cEEG, are crucial as EEG is a resource that requires workforce and hardware, which are not always readily available (71). Using rapid and reduced EEG will also alleviate the burden of staff shortages and reliably provide much-needed EEG services while supporting EEG technologists to avoid burnout and feelings of underappreciation (35). Using ambulatory EEG (18) and smartphone videos for diagnostic purposes will also help triage patients, properly utilize our EMUs, and determine if further testing is necessary for evaluating epileptic and non-epileptic seizures (60–62).

**Figure 1:**
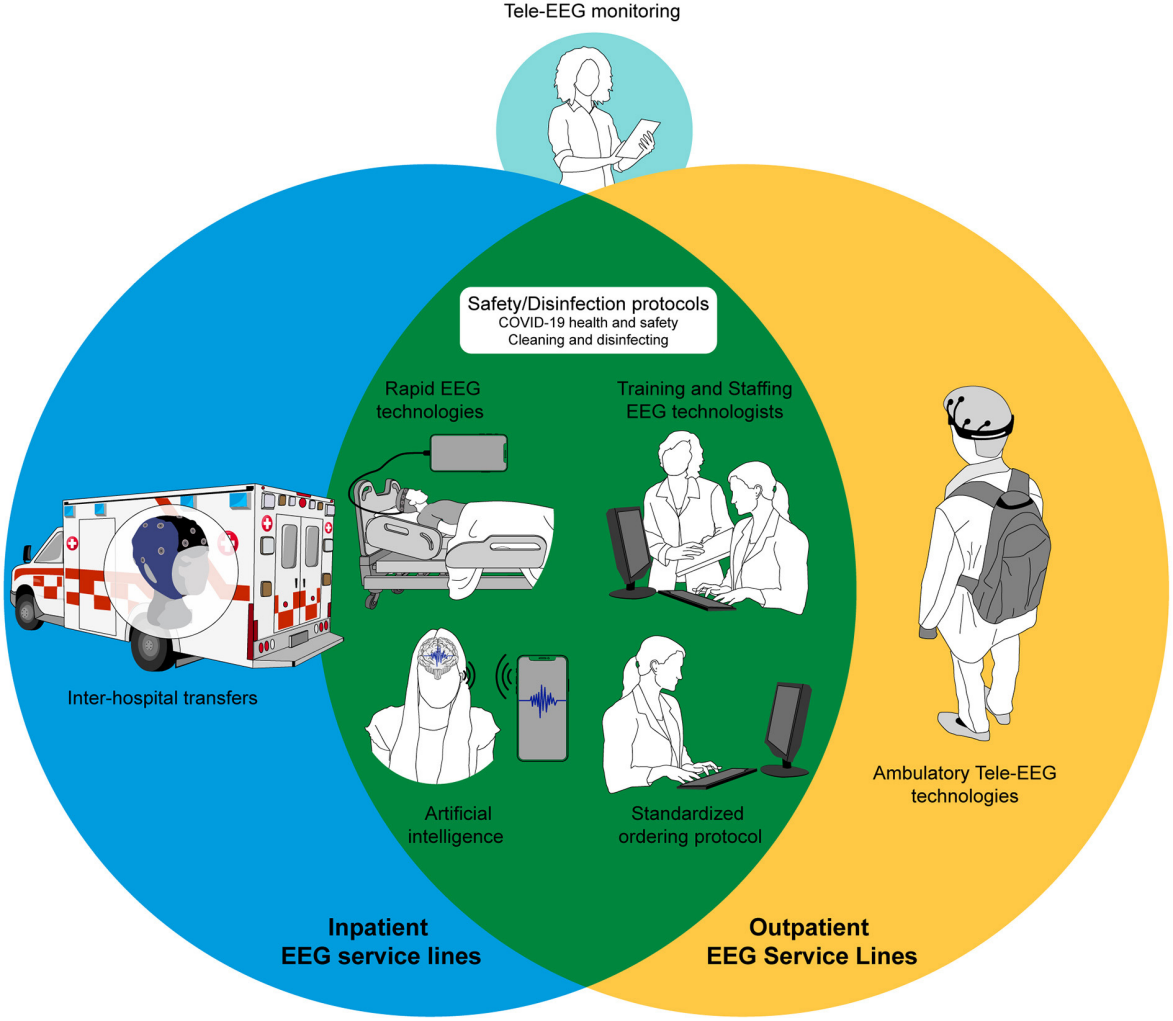
Illustration showing components for an ideal pandemic-proof EEG service line.

When considering our failures as well as achievements during the pandemic, and in preparation for other emergencies unforeseen in the future, an efficient and well-supported EEG service line should be developed at each epilepsy center. Figure 1 provides an example of an ideal future pandemic-proof EEG service line.

## Conclusions

The COVID-19 Pandemic has driven changes in our healthcare system due to the need to adapt to a new resource-limited environment and provide services where they are perceived to be most needed. However, patients with COVID-19-associated conditions and chronic diseases that may not have been deemed urgent or life-threatening suffered due to this prioritization of specific COVID-19 initiatives. Patients with seizures and epilepsy were one of the groups which bore the brunt of these adjustments in healthcare delivery. There have been direct and indirect impacts on epilepsy care, some of which are still to be felt and determined. In particular, EEG and LTVEM have been affected in the outpatient and inpatient settings. Although there were definite tribulations, some of the successes and advancements, including novel approaches to providing neurophysiological and neurodiagnostic services to patients with seizures, are exciting. We hope to make the best out of the pandemic by learning and improving our practices as we move forward. However, we must continue monitoring the pandemic's effects to understand how to prevent catastrophic limitations in EEG services in the future. We must continue innovating to establish an ideal pandemic-proof EEG service line.

## Author contributions

All authors listed have made a substantial, direct, and intellectual contribution to the work and approved it for publication.

## Conflict of interest

The authors declare that the research was conducted in the absence of any commercial or financial relationships that could be construed as a potential conflict of interest.

## Publisher's note

All claims expressed in this article are solely those of the authors and do not necessarily represent those of their affiliated organizations, or those of the publisher, the editors and the reviewers. Any product that may be evaluated in this article, or claim that may be made by its manufacturer, is not guaranteed or endorsed by the publisher.

## Footnotes

^1^https://www.acns.org/practice/covid-19-resources (accessed November 08, 2022).
